# The information-giving skills of resident physicians: relationships with confidence and simulated patient satisfaction

**DOI:** 10.1186/s12909-017-0875-6

**Published:** 2017-02-08

**Authors:** Hirono Ishikawa, Daisuke Son, Masato Eto, Kiyoshi Kitamura, Takahiro Kiuchi

**Affiliations:** 1 Department of Health Communication, School of Public Health, The University of Tokyo, 7-3-1, Hongo, Bunkyo-ku, Tokyo, 113-8655 Japan; 2 International Research Center for Medical Education, Graduate School of Medicine, The University of Tokyo, Tokyo, Japan; 3 General Education Center, University of Tokyo Hospital, Tokyo, Japan; 4 Present Address: International University of Health and Welfare, Chiba, Japan

**Keywords:** Information giving, Communication skills training, Postgraduate education, Residents, the RIAS

## Abstract

**Background:**

Sharing information is crucial for discussion of problems and treatment decision making by patients and physicians. However, the focus of communication skills training in undergraduate medical education has been on building the relationship and gathering information; thus, resident physicians tend to be less confident about sharing information and planning treatment.

This study evaluated the medical interviews conducted by resident physicians with a focus on information giving, and investigated its relationships with their confidence in communication and simulated patient (SP) satisfaction.

**Methods:**

Among 137 junior resident physicians at a university hospital in Japan who participated in a survey of communication skills, 25 volunteered to conduct simulated medical interviews. The medical interviews were video-recorded and analyzed using the Roter Interaction Analysis System, together with additional coding to explore specific features of information giving. The SPs evaluated their satisfaction with the medical interview.

**Results:**

Resident physicians who were more confident in their communication skills provided more information to the patients, while SP satisfaction was associated only with patient-prompted information giving. SPs were more satisfied when the physicians explained the rationales for their opinions and recommendations.

**Conclusion:**

Our findings underscore the importance of providing relevant information in response to the patient requests, and explaining the rationales for the opinions and recommendations. Further investigation is needed to clinically confirm our findings and develop an appropriate communication skills training program.

## Background

Explaining illness is one of the most important, yet most challenging, communication tasks in the practice of health care [1]. Numerous studies have indicated that patients are dissatisfied with the lack of explanation of their conditions. Traditionally, physicians tend to underestimate patients’ demand for information [2], and overestimate the quantity, completeness, and effectiveness of the explanations they provide [3].

Although patients’ desire for as much information as possible is widely acknowledged [4–6], few studies have explored the meaning of “much information.” This lack of clarity can cause a dilemma for physicians in terms of providing full information without overwhelming patients [7]. In their review of the measurement of explanation and information giving during medical consultations, Tuckett and Williams [8] indicated that previous studies have focused on examining the way in which information is given, rather than the information itself. In investigating the information, previous studies typically assessed information giving by simply enumerating physicians’ information-giving statements. Some of such studies reported that the amount of information imparted by physicians was associated positively with patient outcomes such as satisfaction, compliance, and recall/understanding [9].

However, patients’ preferences for information, the knowledge, the ability to understand and utilize information (i.e., health literacy), and information seeking behavior during medical interviews vary a great deal according to individual, which in turn may influence physicians’ information giving and patients’ perception of it [10–13]. In fact, some studies of the total frequency of physicians’ information giving have failed to find significant associations with patients’ perceptions of physicians’ informativeness and patient satisfaction [14–17]. Determining a specific patient’s views, needs, and expectations facilitates provision of information that meets his/her needs to a greater degree than does routinely providing vast amounts of standardized information [18, 19]. A few previous studies attempted to distinguish between information giving initiated by the physician and prompted by the patient (i.e., provided in response to the patient’s question, assertiveness, or expression of concern) [10, 20]. Such a distinction may reflect whether the information giving was responsive to the needs of the patient.

One of the most frequently used quantitative methods for medical interview coding is the Roter Interaction Analysis Systems (RIAS) [21]. The RIAS categorizes information-giving statements according to topic (i.e., medical condition, therapeutic regimen, lifestyle, psychosocial issues, and other information). In addition to topic-based categorization, Tuckett and Williams [8] proposed that information content could be categorized by its depth (e.g., interpretive or “making plain,” and reason-giving or “accounting for”). Qualitative studies have found that physicians explain their diagnostic reasoning to be accountable to patients for their diagnostic conclusions [22, 23]. Sharing the rationale for a diagnosis and treatment recommendation is considered to be an important step for collaborative management [24].

With the increasing focus on communication skills in medical education, many medical schools in Japan have undergone major curriculum reform and now provide lectures on and practice in medical interviewing. However, the focus of communication skills training in undergraduate medical education is on those skills related to building the relationship and gathering information; thus, resident physicians tend to be less confident about sharing information and planning treatment [25]. Training in communication skills for information sharing is needed during residency programs, which should be developed based on evidence related to the provision of high-quality information. However, few studies have empirically shown the specific profiles of successful information giving and the relationship of the confidence with actual communication during the medical interview.

This study aimed to evaluate the communication profiles of medical interviews conducted by junior resident physicians, with particular attention to physicians’ information giving, and to investigate its relationships with their confidence in communication skills and simulated patient (SP) satisfaction. More specifically, we tested the following hypotheses.

1. Physicians with higher confidence in medical interview provide greater amount of information.
2. Physician information giving prompted by patient, rather than self-initiated, is related to higher SP satisfaction.
3. Physician explanation of the rationale for their opinions and recommendations is related to higher SP satisfaction.

## Methods

### Participants

Participants were resident physicians who entered the junior residency program at a university hospital in Tokyo in 2013 and 2014. They were invited to participate in a questionnaire survey during the orientation sessions at the beginning of the residency program [25]. Among 137 survey participants, 25 resident physicians volunteered to participate in the simulated medical interview for this study.

Residents were informed orally and in writing that participation was voluntary and would not influence their evaluation in the residency program. Each participant in the simulated medical interview received an Amazon gift certificate of ¥1000 (approximately US$10) in return. The Institutional Review Board of the Graduate School of Medicine, the University of Tokyo, approved the study procedure (10382).

### Simulated medical interview

Each resident was asked to conduct a medical interview with an SP for about 10 min. A single case scenario was performed by eight voluntary SPs (one male and seven females), who were experienced in acting as SPs for medical education. To ensure the quality of their performance, a manual and evaluation sheets were prepared. In training sessions before the simulated medical interviews, the manual was carefully read and discussed with the participating SPs, who then practiced the scenario with one of the authors, who acted as a resident physician.

The scenario involved a patient who visited a physician to receive the results of an oral glucose tolerance test taken during the previous week. The patient took the test because at a health checkup he/she had been advised to make a medical visit because of a high blood glucose level, although he/she had no subjective symptom. Residents were asked to explain the results of the test and discuss treatment plans. The medical interviews were video-recorded.

## Measures

### Physician confidence in the medical interview

Physicians’ confidence in conducting patient-centered medical interviews was assessed using the self-reported Physician Confidence in the Medical Interview (PCMI) scale [25], administered during the orientation sessions prior to the simulated medical interviews. The scale consists of 21 specific behavioral objectives under seven communication tasks in the medical interview: 1) initiating the session, 2) gathering information, 3) sharing information, 4) planning, 5) closing the session, 6) providing a structure, and 7) building a relationship. In addition, one item assesses physicians’ overall confidence in conducting a medical interview that would be considered satisfactory and acceptable by the patient. Each item was rated on a 4-point Likert scale ranging from 1 (not confident at all) to 4 (very confident). The total score was calculated as the mean of the 22 item scores, with higher scores indicating greater confidence in communicating with the patient (Cronbach’s *α* = 0.98; theoretical range, 1–4). The score was divided at the median and used as a dichotomous variable in subsequent analyses.

### SP satisfaction with physician communication

Immediately upon completion of each simulated medical interview, the SP evaluated the communication skills of the resident with regard to the following five items; whether the physician 1) showed interest in my ideas about my health, 2) understood my main health concerns, 3) listened to me carefully, 4) gave me as much information as I wanted, and 5) talked in terms I could understand. Each item was rated on a 4-point scale ranging from 1 (poor) to 4 (excellent). The total score was calculated as the mean of the five item scores, with higher scores indicating better performance (Cronbach’s *α* = 0.91; theoretical range, 1–4). The score was divided at the median and used as a dichotomous variable in subsequent analyses.

### The process of information giving

The recorded medical interviews were analyzed using the RIAS. The RIAS manual has been translated into Japanese and demonstrated to be a valid and reliable instrument for the analysis of Japanese medical communication [15]. According to the RIAS, the “gives information” category includes “statements that do not explicitly direct the other’s behavior. These statements are characterized by content presented in a neutral manner and/or information regarding actions to be initiated by the speaker or others (e.g., clinic or hospital personnel)” [26]. For the purpose of this study, two additional codings were added to the “gives information” category in the RIAS. First, we categorized physician information giving statements as 1) self-initiated by the physician or 2) prompted by the patient. Prompted information giving was defined as information giving by the physician in response to prior patient statements—including questions, emotional expressions, and information giving—whereas self-initiated information giving was spontaneous provision of information by the physician that was unrelated to previous patient statements. Coding was performed using the block function in the RIAS coding software, which allows marking of specific sequential utterances. Physician-initiated talk and patient-prompted talk were coded as different blocks, and the number of instances of physician information giving in the blocks was counted.

Secondly, in addition to the original RIAS categories, a tag was created for physician information giving statements that provide the rationales for their explanation or advice. The total number of tagged statements was then counted.

The reliability of the coding was assessed over 20 randomly selected consultations, which were coded independently by two coders. Intra-class correlation coefficients (*R* values) were calculated as measures of inter-coder reliability for the communication indicators used in this study. The average *R* value for the RIAS clusters reported in this study was 0.91 (range, 0.85–0.95). The *R* values for the additional coding of self-initiated/prompted information giving were 0.97 and 0.91, respectively, and that of rationale giving was 0.92. Thus, the inter-coder reliability was considered to be adequate.

### Statistical analysis

A *t*-test was used to examine differences in the characteristics of participants in this study and those in the original survey study. Physician information-giving measures were compared between physicians with high and low confidence and between those with high and low SP satisfaction using the Wilcoxon rank-sum test. Statistical analyses were conducted using the Stata 14.1 software (Stata Corporation, TX).

## Results

### Participants’ characteristics

Table 1 presents the characteristics of the participants in this study, with those of the resident physicians participating in the original survey study serving as a reference. A total of 13 male and 12 female residents participated in the simulated medical interviews. PCMI scores did not differ significantly between the participants in this study and those in the original survey study.

**Table 1:** Participants’ characteristics

	Participants of this study				Other participants of the survey study			
	(*N* = 25)				(*N* = 112)			
	Mean	SD	Min	Max	Mean	SD	Min	Max
Age	26.2	2.9	24	36	25.9	3.3	24	41
Gender	N	%			N	%		
Male	13	52.0			63	56.3		
Female	12	48.0			48	42.9		
Missing	0	0.0			1	0.9		
PCMI	2.35	0.76	1.00	4.00	2.40	0.51	1.19	3.86
SP satisfaction	3.42	0.61	2.00	4.00				

### Descriptive results for physician information giving

Descriptive results for physician information giving are shown in Table 2. Based on the RIAS coding, approximately one-third of the physician talk was devoted to information giving, most of which was regarding medical conditions and therapeutic regimens. The mean proportions of patient-prompted and self-initiated physician information giving were similar but varied widely among physicians (48.3% [12.9 to 83.3%] vs. 51.7% [16.7 to 87.1%]). All but one physician made at least one information-giving statement to explain the rationales for their opinions and treatment recommendations (median, 6). Rationale giving comprised 16.0% of the total physician information giving.

**Table 2:** Descriptive results of physician information giving

	Mean	SD	Min	Max
**Information giving**	**42.6**	**12.0**	**23**	**62**
Medical condition	32.2	10.6	13	53
Therapeutic regimen	10.4	5.1	2	20
Lifestyle/psychosocial issues	0.04	0.2	0	1
Patient-prompted	21.0	11.6	6	42
Physician-initiated	21.6	10.4	7	54
Gives rationale	6.5	3.8	0	14
**Total statements in the interview**	**122.6**	**33.3**	**79**	**238**

### Differences in physician information giving according to physician confidence and SP satisfaction

As shown in Table 3, physicians’ confidence in medical interviews was related to their total amount of information giving. Those with higher confidence were likely to provide more information to patients. On the other hand, SP satisfaction was associated with patient-prompted information giving, but not with physician-initiated information giving. That is, patients were satisfied when the physicians gave more information in response to their prompts.

**Table 3:** Differences in physician information giving by physician confidence and SP satisfaction

	PCM					SP satisfaction				
	High		Low		*p*-value	High		Low		*p*-value
	(*N* = 12)		(*N* = 13)			(*N* = 14)		(*N* = 11)		
	Mean	SD	Mean	SD		Mean	SD	Mean	SD	
Total physician information giving	48.08	8.21	37.54	12.91	0.011	46.07	11.29	38.18	11.81	0.247
Patient-prompted	23.00	10.52	19.15	12.67	0.314	25.21	11.44	15.64	9.86	0.038
Physician-initiated	25.08	13.26	18.38	5.82	0.164	20.86	12.15	22.55	8.26	0.298
Giving rationales	8.00	4.18	5.15	2.88	0.081	8.00	3.80	4.64	2.91	0.042

Physicians with higher confidence tended to provide more information to explain the rationales for their opinions and recommendations, although this difference was not statistically significant. SP satisfaction was significantly associated with the frequency of physicians’ provision of information to explain their rationales. SPs were more satisfied with medical interviews in which physicians provided rationales.

## Discussion

This study explored physicians’ information giving in simulated medical interviews, using the RIAS and additional coding, and examined its relationships with physicians’ confidence in the medical interview and SP satisfaction with physician communication.

In the simulated medical interviews, the overall physician communication profile, analyzed by the RIAS, reflected the nature of the scenario. Physicians focused more on providing information and counseling to patients and less on gathering information than found in previous Japanese studies involving routine medical visits of patients with chronic conditions [27, 28]. Also, physicians provided information primarily about medical conditions and therapeutic regimens; few utterances were coded as related to lifestyle/psychosocial issues. This behavior was due in part to the scenario, which involved patients with high blood glucose levels; thus, discussion of diet and exercise was coded as “therapeutic regimen,” not as “lifestyle,” according to the RIAS definition.

Further coding revealed that about half of physician information giving was prompted by the patients, but this proportion varied widely among physicians. Information can be considered to be a source of power to control uncertainty and support dominance and subordination in medical encounters [3]. Typically, the provision of information has been assumed to reflect a physician’s willingness to share decision-making with the patient, comprising more patient-centered communication behavior [29]. However, information giving, especially physician-initiated information giving, can be used to demonstrate the physician’s expertise to the patient, and to control the discussion and decision-making. The results of this study suggest that patients find information provided by physicians in response to their requests to be more informative and satisfying. Our findings underscore the importance of providing appropriate and relevant information based on patient needs as suggested in previous studies [18].

Resident physicians who were more confident in their communication skills provided more information to the patients. This finding suggests that the measure of physicians’ confidence in medical interviews used in this study reflects their actual communication skills. This finding is generally consistent with a previous report that experienced physicians provided more information than did less-experienced physicians [10]. Interestingly, however, physician confidence was not associated with whether the information was prompted by the patient, which was associated significantly with SP satisfaction. It has been noted that physicians’ patient-centered attitudes might decline during medical education [30]. Further examination of whether and how the proportion of patient-prompted information giving in relation to physician-initiated information giving changes according to confidence in communication and clinical experience is warranted.

Furthermore, our findings suggest the importance of providing rationales for decisions. Physicians with higher confidence in their communication skills tended to explain the rationales for their opinions and recommendations, which was associated with greater SP satisfaction. It has been suggested that patients’ adherence to management plans is often poor because physicians seldom explain their rationales in any detail or provide explanations related to the patients’ illness framework [24]. Sharing of thought processes and rationales by physicians would facilitate collaborative management.

### Limitations

Several limitations should be noted when interpreting the findings of this study. First, this study was preliminary, with a small sample, which may limit the generalizability of the findings. The sample used was part of a larger study of resident physicians, and the characteristics of physicians in this sample did not differ from those of physicians in the original sample. Although the sample was considered to be reasonable for a preliminary investigation, the findings should be confirmed in a larger study. Secondly, the use of SPs may have created an artificial phenomenon, although the SPs in this study were not “standardized” patients, unlike the SPs in the Objective Structured Clinical Examinations, and their responses to physicians’ communication were more spontaneous, reflecting the responses of a real patient. Also, it was necessary to investigate physicians’ information giving while controlling for other influencing factors, such as the severity of the patient’s condition. Thus, although it was a pilot approach, this study sheds light on the specific profiles of successful information giving. Further study is needed to clinically confirm our findings with real patients in different contexts and extend them to patient health outcomes, as well as to develop a communication skills training program for medical education.

## Conclusions

This study explored the profiles of physicians’ information giving using the RIAS, together with additional coding. About half of physician information giving was prompted by patients, but this proportion varied markedly among the physicians. Resident physicians who were more confident in their communication skills provided more information to the patients, while simulated patient satisfaction was associated with the amount of patient-prompted rather than physician-initiated information. Providing the information in response to patients’ story and requests might be important for effective information giving. Also, patients may find the information explaining the rationales for physicians’ opinions and recommendations helpful. Further investigation is needed to clinically confirm our findings and develop an appropriate communication skills training program for resident physicians.
